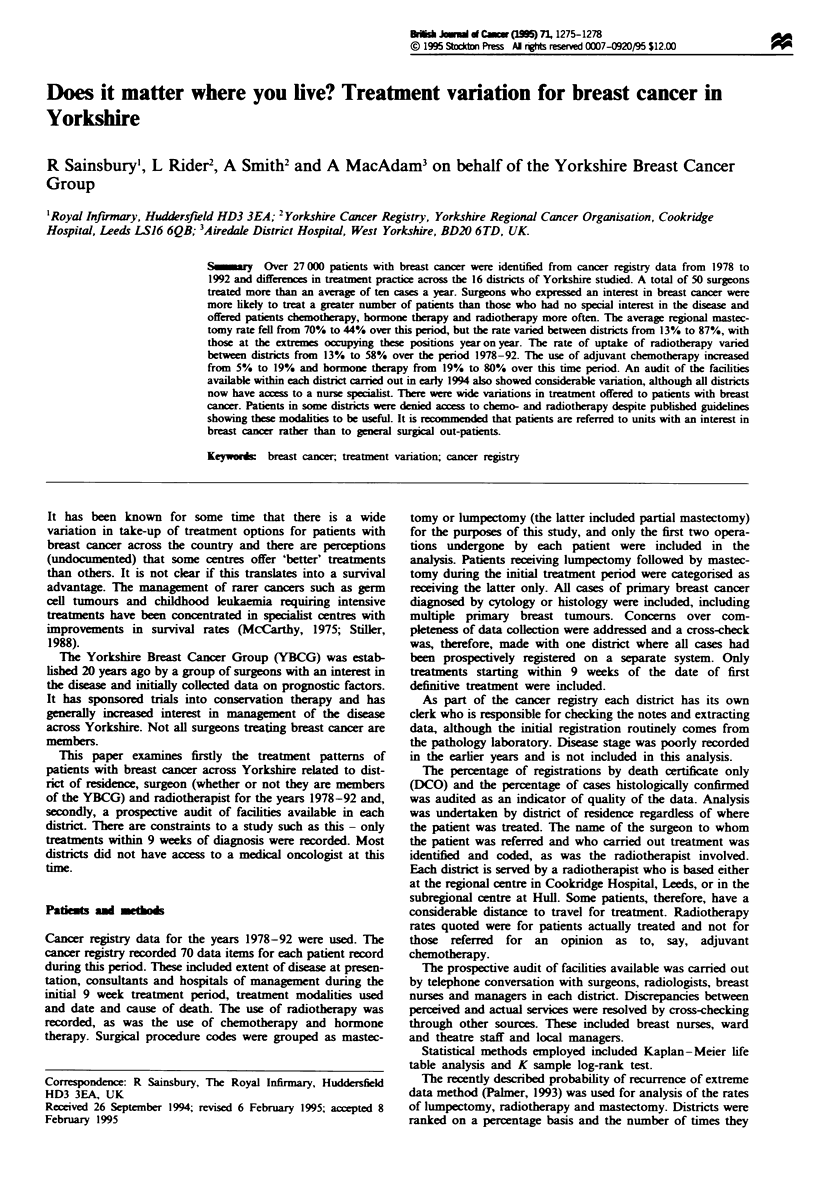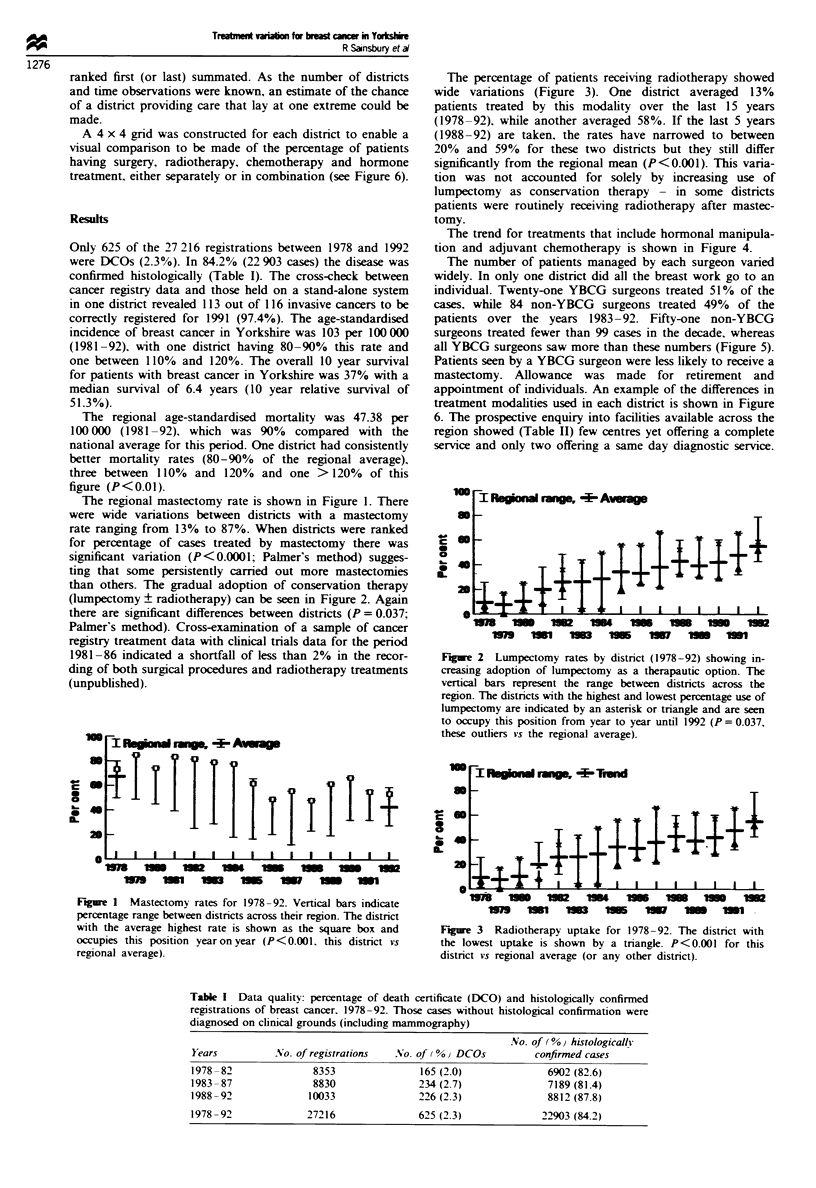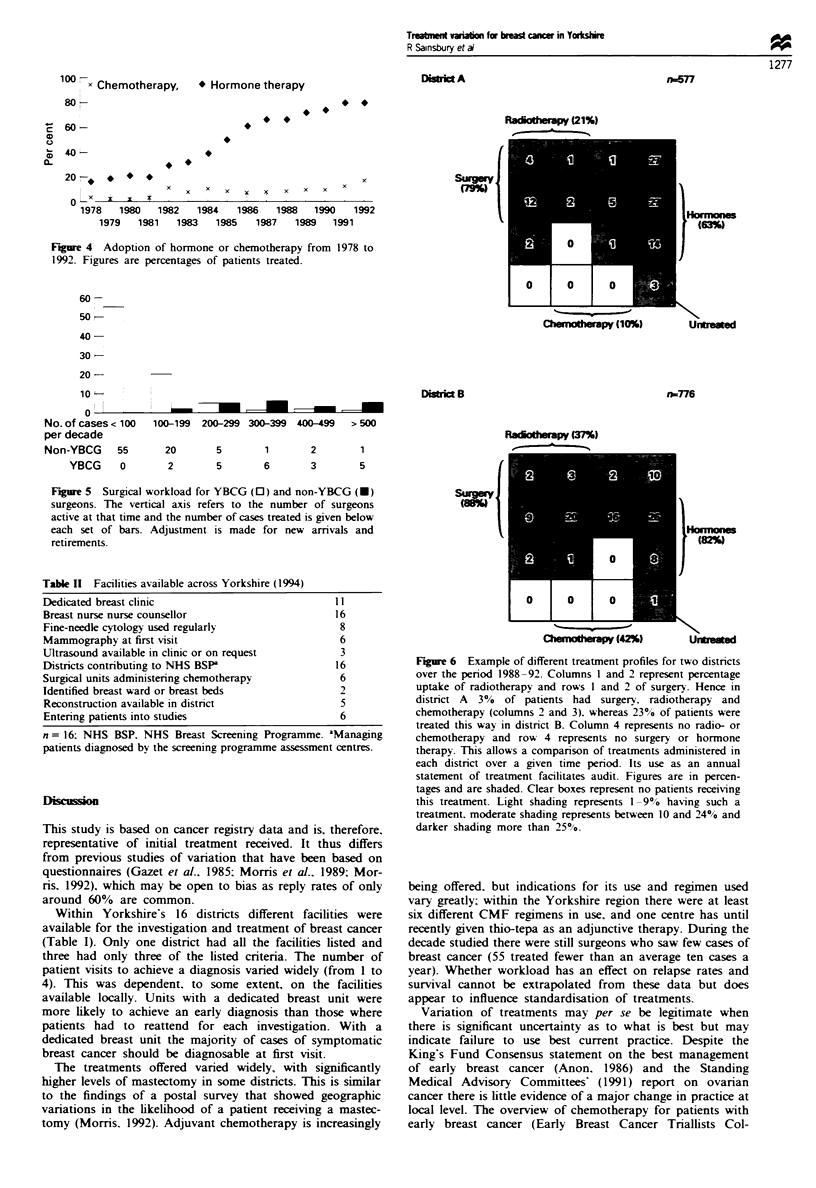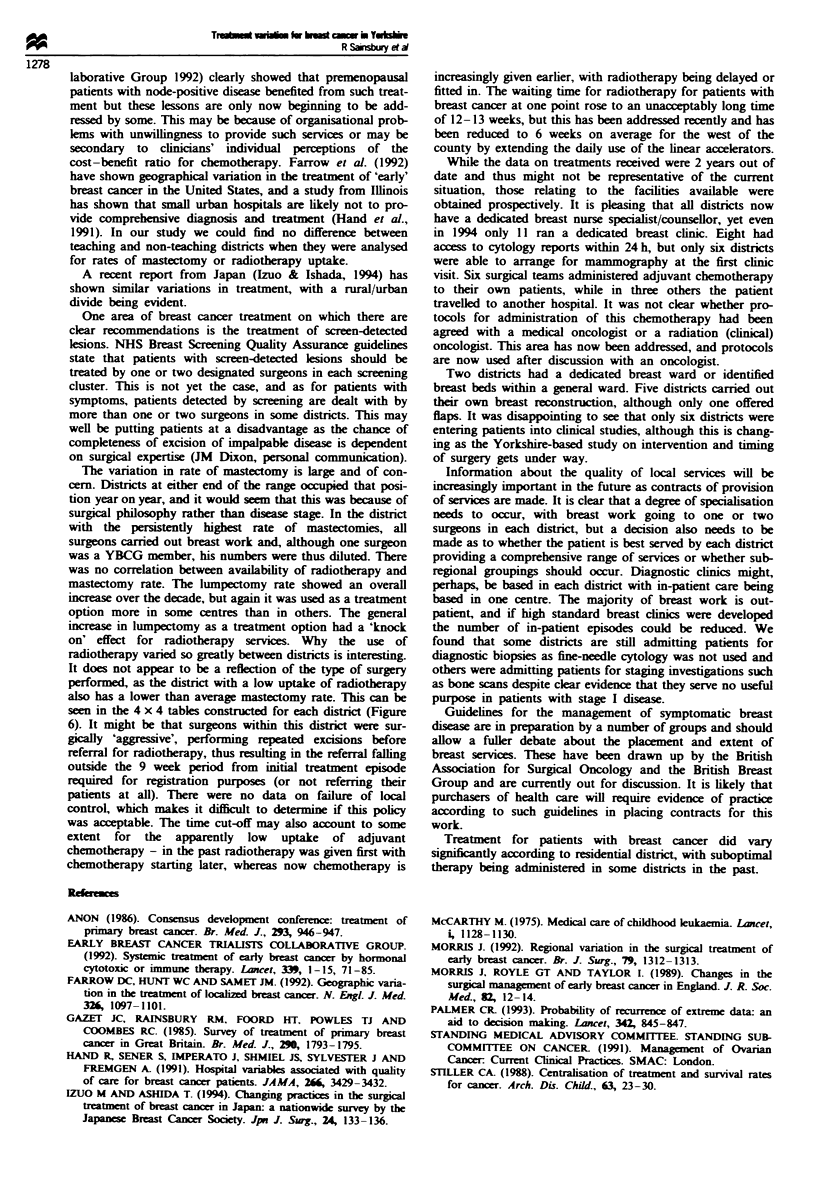# Does it matter where you live? Treatment variation for breast cancer in Yorkshire. The Yorkshire Breast Cancer Group.

**DOI:** 10.1038/bjc.1995.246

**Published:** 1995-06

**Authors:** R. Sainsbury, L. Rider, A. Smith, A. MacAdam

**Affiliations:** Royal Infirmary, Huddersfield, UK.

## Abstract

Over 27,000 patients with breast cancer were identified from cancer registry data from 1978 to 1992 and differences in treatment practice across the 16 districts of Yorkshire studied. A total of 50 surgeons treated more than an average of ten cases a year. Surgeons who expressed an interest in breast cancer were more likely to treat a greater number of patients than those who had no special interest in the disease and offered patients chemotherapy, hormone therapy and radiotherapy more often. The average regional mastectomy rate fell from 70% to 44% over this period, but the rate varied between districts from 13% to 87%, with those at the extremes occupying these positions year on year. The rate of uptake of radiotherapy varied between districts from 13% to 58% over the period 1978-92. The use of adjuvant chemotherapy increased from 5% to 19% and hormone therapy from 19% to 80% over this time period. An audit of the facilities available within each district carried out in early 1994 also showed considerable variation, although all districts now have access to a nurse specialist. There were wide variations in treatment offered to patients with breast cancer. Patients in some districts were denied access to chemo- and and radiotherapy despite published guidelines showing these modalities to be useful. It is recommended that patients are referred to units with an interest in breast cancer rather than to general surgical out-patients.


© 1995 Stockton Press All rights reserved 0007-0920/95 $12.00

### Does it matter where you live? Treatment variation for breast cancer in Yorkshire

R Sainsbury[1], L Rider[2], A Smith[2] and A MacAdam[3] on behalf of the Yorkshire Breast Cancer Group

[1]*Royal Infirmary, Huddersfield HD3 3EA;* [2]*Yorkshire Cancer Registry, Yorkshire Regional Cancer Organisation, Cookridge Hospital, Leeds LS16 6QB;* [3]*Airedale District Hospital, West Yorkshire, BD20 6TD, UK.*

**Summary** Over 27 000 patients with breast cancer were identified from cancer registry data from 1978 to 1992 and differences in treatment practice across the 16 districts of Yorkshire studied. A total of 50 surgeons treated more than an average of ten cases a year. Surgeons who expressed an interest in breast cancer were more likely to treat a greater number of patients than those who had no special interest in the disease and offered patients chemotherapy, hormone therapy and radiotherapy more often. The average regional mastectomy rate fell from 70% to 44% over this period, but the rate varied between districts from 13% to 87%, with those at the extremes occupying these positions year on year. The rate of uptake of radiotherapy varied between districts from 13% to 58% over the period 1978-92. The use of adjuvant chemotherapy increased from 5% to 19% and hormone therapy from 19% to 80% over this time period. An audit of the facilities available within each district carried out in early 1994 also showed considerable variation, although all districts now have access to a nurse specialist. There were wide variations in treatment offered to patients with breast cancer. Patients in some districts were denied access to chemo- and radiotherapy despite published guidelines showing these modalities to be useful. It is recommended that patients are referred to units with an interest in breast cancer rather than to general surgical out-patients.

**Keywords:** breast cancer; treatment variation; cancer registry

It has been known for some time that there is a wide variation in take-up of treatment options for patients with breast cancer across the country and there are perceptions (undocumented) that some centres offer 'better' treatments than others. It is not clear if this translates into a survival advantage. The management of rarer cancers such as germ cell tumours and childhood leukaemia requiring intensive treatments have been concentrated in specialist centres with improvements in survival rates (McCarthy, 1975; Stiller, 1988).

The Yorkshire Breast Cancer Group (YBCG) was established 20 years ago by a group of surgeons with an interest in the disease and initially collected data on prognostic factors. It has sponsored trials into conservation therapy and has generally increased interest in management of the disease across Yorkshire. Not all surgeons treating breast cancer are members.

This paper examines firstly the treatment patterns of patients with breast cancer across Yorkshire related to district of residence, surgeon (whether or not they are members of the YBCG) and radiotherapist for the years 1978-92 and, secondly, a prospective audit of facilities available in each district. There are constraints to a study such as this - only treatments within 9 weeks of diagnosis were recorded. Most districts did not have access to a medical oncologist at this time.

### Patients and methods

Cancer registry data for the years 1978-92 were used. The cancer registry recorded 70 data items for each patient record during this period. These included extent of disease at presentation, consultants and hospitals of management during the initial 9 week treatment period, treatment modalities used and date and cause of death. The use of radiotherapy was recorded, as was the use of chemotherapy and hormone therapy. Surgical procedure codes were grouped as mastectomy or lumpectomy (the latter included partial mastectomy) for the purposes of this study, and only the first two operations undergone by each patient were included in the analysis. Patients receiving lumpectomy followed by mastectomy during the initial treatment period were categorised as receiving the latter only. All cases of primary breast cancer diagnosed by cytology or histology were included, including multiple primary breast tumours. Concerns over completeness of data collection were addressed and a cross-check was, therefore, made with one district where all cases had been prospectively registered on a separate system. Only treatments starting within 9 weeks of the date of first definitive treatment were included.

As part of the cancer registry each district has its own clerk who is responsible for checking the notes and extracting data, although the initial registration routinely comes from the pathology laboratory. Disease stage was poorly recorded in the earlier years and is not included in this analysis.

The percentage of registrations by death certificate only (DCO) and the percentage of cases histologically confirmed was audited as an indicator of quality of the data. Analysis was undertaken by district of residence regardless of where the patient was treated. The name of the surgeon to whom the patient was referred and who carried out treatment was identified and coded, as was the radiotherapist involved. Each district is served by a radiotherapist who is based either at the regional centre in Cookridge Hospital, Leeds, or in the subregional centre at Hull. Some patients, therefore, have a considerable distance to travel for treatment. Radiotherapy rates quoted were for patients actually treated and not for those referred for an opinion as to, say, adjuvant chemotherapy.

The prospective audit of facilities available was carried out by telephone conversation with surgeons, radiologists, breast nurses and managers in each district. Discrepancies between perceived and actual services were resolved by cross-checking through other sources. These included breast nurses, ward and theatre staff and local managers.

Statistical methods employed included Kaplan-Meier life table analysis and $K$ sample log-rank test.

The recently described probability of recurrence of extreme data method (Palmer, 1993) was used for analysis of the rates of lumpectomy, radiotherapy and mastectomy. Districts were ranked on a percentage basis and the number of times they

Correspondence: R Sainsbury, The Royal Infirmary, Huddersfield HD3 3EA, UK
Received 26 September 1994; revised 6 February 1995; accepted 8 February 1995

ranked first (or last) summated. As the number of districts and time observations were known, an estimate of the chance of a district providing care that lay at one extreme could be made.

A 4 × 4 grid was constructed for each district to enable a visual comparison to be made of the percentage of patients having surgery, radiotherapy, chemotherapy and hormone treatment, either separately or in combination (see Figure 6).

### Results

Only 625 of the 27 216 registrations between 1978 and 1992 were DCOs (2.3%). In 84.2% (22 903 cases) the disease was confirmed histologically (Table I). The cross-check between cancer registry data and those held on a stand-alone system in one district revealed 113 out of 116 invasive cancers to be correctly registered for 1991 (97.4%). The age-standardised incidence of breast cancer in Yorkshire was 103 per 100 000 (1981–92), with one district having 80–90% this rate and one between 110% and 120%. The overall 10 year survival for patients with breast cancer in Yorkshire was 37% with a median survival of 6.4 years (10 year relative survival of 51.3%).

The regional age-standardised mortality was 47.38 per 100 000 (1981–92), which was 90% compared with the national average for this period. One district had consistently better mortality rates (80–90% of the regional average), three between 110% and 120% and one >120% of this figure ($P<0.01$).

The regional mastectomy rate is shown in Figure 1. There were wide variations between districts with a mastectomy rate ranging from 13% to 87%. When districts were ranked for percentage of cases treated by mastectomy there was significant variation ($P<0.0001$; Palmer's method) suggesting that some persistently carried out more mastectomies than others. The gradual adoption of conservation therapy (lumpectomy ± radiotherapy) can be seen in Figure 2. Again there are significant differences between districts ($P=0.037$; Palmer's method). Cross-examination of a sample of cancer registry treatment data with clinical trials data for the period 1981–86 indicated a shortfall of less than 2% in the recording of both surgical procedures and radiotherapy treatments (unpublished).

**Figure 1** Mastectomy rates for 1978–92. Vertical bars indicate percentage range between districts across their region. The district with the average highest rate is shown as the square box and occupies this position year on year ($P<0.001$, this district *vs* regional average).

The percentage of patients receiving radiotherapy showed wide variations (Figure 3). One district averaged 13% patients treated by this modality over the last 15 years (1978–92), while another averaged 58%. If the last 5 years (1988–92) are taken, the rates have narrowed to between 20% and 59% for these two districts but they still differ significantly from the regional mean ($P<0.001$). This variation was not accounted for solely by increasing use of lumpectomy as conservation therapy – in some districts patients were routinely receiving radiotherapy after mastectomy.

The trend for treatments that include hormonal manipulation and adjuvant chemotherapy is shown in Figure 4.

The number of patients managed by each surgeon varied widely. In only one district did all the breast work go to an individual. Twenty-one YBCG surgeons treated 51% of the cases, while 84 non-YBCG surgeons treated 49% of the patients over the years 1983–92. Fifty-one non-YBCG surgeons treated fewer than 99 cases in the decade, whereas all YBCG surgeons saw more than these numbers (Figure 5). Patients seen by a YBCG surgeon were less likely to receive a mastectomy. Allowance was made for retirement and appointment of individuals. An example of the differences in treatment modalities used in each district is shown in Figure 6. The prospective enquiry into facilities available across the region showed (Table II) few centres yet offering a complete service and only two offering a same day diagnostic service.

**Figure 2** Lumpectomy rates by district (1978–92) showing increasing adoption of lumpectomy as a therapautic option. The vertical bars represent the range between districts across the region. The districts with the highest and lowest percentage use of lumpectomy are indicated by an asterisk or triangle and are seen to occupy this position from year to year until 1992 ($P=0.037$, these outliers *vs* the regional average).

**Figure 3** Radiotherapy uptake for 1978–92. The district with the lowest uptake is shown by a triangle. $P<0.001$ for this district *vs* regional average (or any other district).

**Table I** Data quality: percentage of death certificate (DCO) and histologically confirmed registrations of breast cancer, 1978–92. Those cases without histological confirmation were diagnosed on clinical grounds (including mammography)

| *Years* | *No. of registrations* | *No. of (%) DCOs* | *No. of (%) histologically confirmed cases* |
|---|---|---|---|
| 1978–82 | 8353 | 165 (2.0) | 6902 (82.6) |
| 1983–87 | 8830 | 234 (2.7) | 7189 (81.4) |
| 1988–92 | 10033 | 226 (2.3) | 8812 (87.8) |
| 1978–92 | 27216 | 625 (2.3) | 22903 (84.2) |

**Figure 4** Adoption of hormone or chemotherapy from 1978 to 1992. Figures are percentages of patients treated.

**Figure 5** Surgical workload for YBCG (□) and non-YBCG (■) surgeons. The vertical axis refers to the number of surgeons active at that time and the number of cases treated is given below each set of bars. Adjustment is made for new arrivals and retirements.

**Table II** Facilities available across Yorkshire (1994)

| | |
|---|---|
| Dedicated breast clinic | 11 |
| Breast nurse nurse counsellor | 16 |
| Fine-needle cytology used regularly | 8 |
| Mammography at first visit | 6 |
| Ultrasound available in clinic or on request | 3 |
| Districts contributing to NHS BSP[a] | 16 |
| Surgical units administering chemotherapy | 6 |
| Identified breast ward or breast beds | 2 |
| Reconstruction available in district | 5 |
| Entering patients into studies | 6 |

*n* = 16; NHS BSP, NHS Breast Screening Programme. [a]Managing patients diagnosed by the screening programme assessment centres.

**Figure 6** Example of different treatment profiles for two districts over the period 1988–92. Columns 1 and 2 represent percentage uptake of radiotherapy and rows 1 and 2 of surgery. Hence in district A 3% of patients had surgery, radiotherapy and chemotherapy (columns 2 and 3), whereas 23% of patients were treated this way in district B. Column 4 represents no radio- or chemotherapy and row 4 represents no surgery or hormone therapy. This allows a comparison of treatments administered in each district over a given time period. Its use as an annual statement of treatment facilitates audit. Figures are in percentages and are shaded. Clear boxes represent no patients receiving this treatment. Light shading represents 1–9% having such a treatment, moderate shading represents between 10 and 24% and darker shading more than 25%.

### Discussion

This study is based on cancer registry data and is, therefore, representative of initial treatment received. It thus differs from previous studies of variation that have been based on questionnaires (Gazet *et al*., 1985; Morris *et al*., 1989; Morris, 1992), which may be open to bias as reply rates of only around 60% are common.

Within Yorkshire's 16 districts different facilities were available for the investigation and treatment of breast cancer (Table I). Only one district had all the facilities listed and three had only three of the listed criteria. The number of patient visits to achieve a diagnosis varied widely (from 1 to 4). This was dependent, to some extent, on the facilities available locally. Units with a dedicated breast unit were more likely to achieve an early diagnosis than those where patients had to reattend for each investigation. With a dedicated breast unit the majority of cases of symptomatic breast cancer should be diagnosable at first visit.

The treatments offered varied widely, with significantly higher levels of mastectomy in some districts. This is similar to the findings of a postal survey that showed geographic variations in the likelihood of a patient receiving a mastectomy (Morris, 1992). Adjuvant chemotherapy is increasingly being offered, but indications for its use and regimen used vary greatly; within the Yorkshire region there were at least six different CMF regimens in use, and one centre has until recently given thio-tepa as an adjunctive therapy. During the decade studied there were still surgeons who saw few cases of breast cancer (55 treated fewer than an average ten cases a year). Whether workload has an effect on relapse rates and survival cannot be extrapolated from these data but does appear to influence standardisation of treatments.

Variation of treatments may *per se* be legitimate when there is significant uncertainty as to what is best but may indicate failure to use best current practice. Despite the King's Fund Consensus statement on the best management of early breast cancer (Anon, 1986) and the Standing Medical Advisory Committees' (1991) report on ovarian cancer there is little evidence of a major change in practice at local level. The overview of chemotherapy for patients with early breast cancer (Early Breast Cancer Triallists Collaborative Group 1992) clearly showed that premenopausal patients with node-positive disease benefited from such treatment but these lessons are only now beginning to be addressed by some. This may be because of organisational problems with unwillingness to provide such services or may be secondary to clinicians' individual perceptions of the cost-benefit ratio for chemotherapy. Farrow *et al.* (1992) have shown geographical variation in the treatment of 'early' breast cancer in the United States, and a study from Illinois has shown that small urban hospitals are likely not to provide comprehensive diagnosis and treatment (Hand *et al.*, 1991). In our study we could find no difference between teaching and non-teaching districts when they were analysed for rates of mastectomy or radiotherapy uptake.

A recent report from Japan (Izuo & Ishada, 1994) has shown similar variations in treatment, with a rural/urban divide being evident.

One area of breast cancer treatment on which there are clear recommendations is the treatment of screen-detected lesions. NHS Breast Screening Quality Assurance guidelines state that patients with screen-detected lesions should be treated by one or two designated surgeons in each screening cluster. This is not yet the case, and as for patients with symptoms, patients detected by screening are dealt with by more than one or two surgeons in some districts. This may well be putting patients at a disadvantage as the chance of completeness of excision of impalpable disease is dependent on surgical expertise (JM Dixon, personal communication).

The variation in rate of mastectomy is large and of concern. Districts at either end of the range occupied that position year on year, and it would seem that this was because of surgical philosophy rather than disease stage. In the district with the persistently highest rate of mastectomies, all surgeons carried out breast work and, although one surgeon was a YBCG member, his numbers were thus diluted. There was no correlation between availability of radiotherapy and mastectomy rate. The lumpectomy rate showed an overall increase over the decade, but again it was used as a treatment option more in some centres than in others. The general increase in lumpectomy as a treatment option had a 'knock on' effect for radiotherapy services. Why the use of radiotherapy varied so greatly between districts is interesting. It does not appear to be a reflection of the type of surgery performed, as the district with a low uptake of radiotherapy also has a lower than average mastectomy rate. This can be seen in the 4 × 4 tables constructed for each district (Figure 6). It might be that surgeons within this district were surgically 'aggressive', performing repeated excisions before referral for radiotherapy, thus resulting in the referral falling outside the 9 week period from initial treatment episode required for registration purposes (or not referring their patients at all). There were no data on failure of local control, which makes it difficult to determine if this policy was acceptable. The time cut-off may also account to some extent for the apparently low uptake of adjuvant chemotherapy – in the past radiotherapy was given first with chemotherapy starting later, whereas now chemotherapy is increasingly given earlier, with radiotherapy being delayed or fitted in. The waiting time for radiotherapy for patients with breast cancer at one point rose to an unacceptably long time of 12–13 weeks, but this has been addressed recently and has been reduced to 6 weeks on average for the west of the county by extending the daily use of the linear accelerators.

While the data on treatments received were 2 years out of date and thus might not be representative of the current situation, those relating to the facilities available were obtained prospectively. It is pleasing that all districts now have a dedicated breast nurse specialist/counsellor, yet even in 1994 only 11 ran a dedicated breast clinic. Eight had access to cytology reports within 24 h, but only six districts were able to arrange for mammography at the first clinic visit. Six surgical teams administered adjuvant chemotherapy to their own patients, while in three others the patient travelled to another hospital. It was not clear whether protocols for administration of this chemotherapy had been agreed with a medical oncologist or a radiation (clinical) oncologist. This area has now been addressed, and protocols are now used after discussion with an oncologist.

Two districts had a dedicated breast ward or identified breast beds within a general ward. Five districts carried out their own breast reconstruction, although only one offered flaps. It was disappointing to see that only six districts were entering patients into clinical studies, although this is changing as the Yorkshire-based study on intervention and timing of surgery gets under way.

Information about the quality of local services will be increasingly important in the future as contracts of provision of services are made. It is clear that a degree of specialisation needs to occur, with breast work going to one or two surgeons in each district, but a decision also needs to be made as to whether the patient is best served by each district providing a comprehensive range of services or whether subregional groupings should occur. Diagnostic clinics might, perhaps, be based in each district with in-patient care being based in one centre. The majority of breast work is outpatient, and if high standard breast clinics were developed the number of in-patient episodes could be reduced. We found that some districts are still admitting patients for diagnostic biopsies as fine-needle cytology was not used and others were admitting patients for staging investigations such as bone scans despite clear evidence that they serve no useful purpose in patients with stage I disease.

Guidelines for the management of symptomatic breast disease are in preparation by a number of groups and should allow a fuller debate about the placement and extent of breast services. These have been drawn up by the British Association for Surgical Oncology and the British Breast Group and are currently out for discussion. It is likely that purchasers of health care will require evidence of practice according to such guidelines in placing contracts for this work.

Treatment for patients with breast cancer did vary significantly according to residential district, with suboptimal therapy being administered in some districts in the past.

### References

ANON (1986). Consensus development conference: treatment of primary breast cancer. *Br. Med. J.*, **293**, 946–947.

EARLY BREAST CANCER TRIALISTS COLLABORATIVE GROUP. (1992). Systemic treatment of early breast cancer by hormonal cytotoxic or immune therapy. *Lancet*, **339**, 1–15, 71–85.

FARROW DC, HUNT WC AND SAMET JM. (1992). Geographic variation in the treatment of localized breast cancer. *N. Engl. J. Med.* **326**, 1097–1101.

GAZET JC, RAINSBURY RM, FOORD HT, POWLES TJ AND COOMBES RC. (1985). Survey of treatment of primary breast cancer in Great Britain. *Br. Med. J.*, **290**, 1793–1795.

HAND R, SENER S, IMPERATO J, SHMIEL JS, SYLVESTER J AND FREMGEN A. (1991). Hospital variables associated with quality of care for breast cancer patients. *JAMA*, **266**, 3429–3432.

IZUO M AND ASHIDA T. (1994). Changing practices in the surgical treatment of breast cancer in Japan: a nationwide survey by the Japanese Breast Cancer Society. *Jpn J. Surg.*, **24**, 133–136.

McCARTHY M. (1975). Medical care of childhood leukaemia. *Lancet*, **i**, 1128–1130.

MORRIS J. (1992). Regional variation in the surgical treatment of early breast cancer. *Br. J. Surg.*, **79**, 1312–1313.

MORRIS J, ROYLE GT AND TAYLOR I. (1989). Changes in the surgical management of early breast cancer in England. *J. R. Soc. Med.*, **82**, 12–14.

PALMER CR. (1993). Probability of recurrence of extreme data: an aid to decision making. *Lancet*, **342**, 845–847.

STANDING MEDICAL ADVISORY COMMITTEE. STANDING SUBCOMMITTEE ON CANCER. (1991). Management of Ovarian Cancer: Current Clinical Practices. SMAC: London.

STILLER CA. (1988). Centralisation of treatment and survival rates for cancer. *Arch. Dis. Child.*, **63**, 23–30.